# Impact of age on the efficacy of bone marrow mononuclear cell transplantation in experimental stroke

**DOI:** 10.1186/2040-7378-4-17

**Published:** 2012-08-24

**Authors:** Daniel-Christoph Wagner, Mitja Bojko, Myriam Peters, Marlene Lorenz, Cornelia Voigt, Alexander Kaminski, Dirk Hasenclever, Markus Scholz, Alexander Kranz, Gesa Weise, Johannes Boltze

**Affiliations:** 1 Fraunhofer Institute for Cell Therapy and Immunology, Leipzig, Germany; 2 Translational Centre for Regenerative Medicine, University of Leipzig, Leipzig, Germany; 3 Department of Urology, University of Leipzig, Leipzig, Germany; 4 Department of Cardiac Surgery, University of Rostock, Rostock, Germany; 5 Institute for Medical Informatics, Statistics & Epidemiology, University of Leipzig, Leipzig, Germany

**Keywords:** Brain ischemia, Cell transplantation, Bone marrow cell transplantation, Age, Comorbidity, SHR

## Abstract

Bone marrow-derived mononuclear cells (BM MNC) have been effectively used to treat experimental stroke. Most of the preclinical trials have been performed in young and healthy laboratory animals, even though age and hypertension are major risk factors for stroke. To determine the influence of age on the properties of BM MNCs after cerebral ischemia, we compared the efficacy of aged and young BM MNC in an in vitro model of cerebral hypoxia and in an adapted in vivo model of stroke. Human BM MNCs were obtained from healthy young or aged donors and either co-cultured with rat hippocampal slices exposed to oxygen glucose deprivation (OGD), or transplanted intravenously 24 h after permanent middle cerebral artery occlusion in aged (18 months) spontaneously hypertensive rats (SHR). Efficacy was examined by quantification of hippocampal cell death, or respectively, by neurofunctional tests and MR investigations. Co-cultivation with young, but not with aged BM MNCs significantly reduced the hippocampal cell death after OGD. Transplantation of both young and old BM MNCs did not reduce functional deficits or ischemic lesion volume after stroke in aged SHR. These results suggest a significant impact of age on the therapeutic efficacy of BM MNCs after cerebral ischemia.

## Background

Cerebral ischemia is one of the leading causes for mortality and disability in industrialized countries, and its treatment is seriously restricted by a tight therapeutic time frame. Less than 5% of stroke patients receive thrombolysis, primarily due to delayed clinical presentation [1]. Consequently, one of the main goals of modern stroke research is an extension of the therapeutic time window. Cell based therapies might fulfil this expectation, since cells are suspected to modulate protective and restorative effects even days after the onset of brain ischemia [2]. Bone marrow derived mononuclear cells (BM MNC) are promising candidates for acute stroke treatment, since these cells can be harvested and re-transplanted acutely in an autologous fashion [3]. An open-label prospective study just recently showed that bone marrow harvest and reinfusion of autologous MNCs in patients with acute middle cerebral artery ischemic stroke is safe and feasible [4].

BM MNC transplantation in experimental models of brain ischemia and spinal cord injury resulted in a significantly improved functional recovery [5-7], and the therapeutic time window for these effects seems to be between 3 h-72 h after stroke onset [8]. However, most of the preclinical trials have been performed in young or middle-aged, healthy laboratory animals - even though age and hypertension are crucial risk factors for stroke [9], and the susceptibility of the ischemic brain for cell-based therapies might be altered with age. Beyond that, age and comorbidities have to be particularly considered if autologous approaches are intended, since not only the receiving tissue but also the donor tissue (i.e. the bone marrow) is subjected to aging processes and might be altered in its functionality. In the present study we aimed to analyze the effect of age on the efficacy of BM MNC transplantation for cerebral ischemia. For this, we investigated the therapeutic efficacy of human BM MNCs obtained from aged or young donors in an ex vivo model of hypoxia-ischemia and in an in vivo model of stroke using aged hypertensive rats.

## Methods

### BM MNC preparation

Cryopreserved human bone marrow derived mononuclear cells (BM MNC) from young donors (n = 4; 24 ± 4 years) were obtained from Lonza (Walkersville, USA). BM MNCs have been isolated by Ficoll-Paque density gradient centrifugation according to the manufacturer’s instructions. Aged bone marrow was collected from healthy elderlies (n = 4; 68 ± 1 years) after informed consent and approval by the ethics review board of the University of Rostock, Germany. Bone marrow cells were layered above Lymphocyte Separation Medium LSM 1077 (PAA Laboratories, Coelbe, Germany) and centrifuged at 800 × g for 20 min. The mononuclear cell layer was removed and washed one time with PBS (400 × g, 10 min). Finally, BM MNCs were resuspended in 10% dimethyl sulfoxide (DMSO; Merck, Hohenbrunn, Germany) and stored in liquid nitrogen. Prior to use, BM MNCs from both sources were thawed rapidly and separated by 75 U/mL Accutase treatment and washed in RPMI (PAA Laboratories, Pasching, Austria).

### Determination of hematopoietic stem cells

The hematopoietic potential of BM MNCs was determined using a methylcellulose based colony forming unit assay. 1.1x10E5/mL cells were mixed with methylcellulose, plated on petri dishes and incubated for 14 days at 37°C in 5% CO_2_ and 95% humidity. The colonies were divided into burst forming units – erythroid (BFU-E) and granulocyte/monocyte colony forming units (CFU-GM) and subsequently counted. The amount and vitality of CD34^+^ cells were quantified by FACSCalibur flow cytometer (Becton Dickinson) using 7-Amino-actinomycin D (7AAD, BD Biosciences), an APC-coupled anti-CD34 and a PC7-coupled anti-CD45 antibody (Beckman Coulter, Krefeld, Germany).

### Organotypic hippocampal slice cultures and oxygen-glucose deprivation

Organotypic hippocampal slice cultures (OHC) were prepared from postnatal Wistar rats (day 8-9, Harlan-Winkelman, Borchen, Germany) as described previously [10]. Animals were sacrificed by decapitation. Hippocampi were dissected and transversally sliced (350 μm) on a McIlwain tissue chopper (The Mickle Laboratory Engineering, Guildford, UK). OHC were transferred to humidified 0.4 μm porous Millicell membranes (Millipore, Morsheim, France) and maintained in 1 mL serum-based medium (50% MEM-Hanks, 25% HBSS, 17 mM HEPES, 5 mM glucose, 1 mM L-glutamin, 25% horse serum, 0.5% gentamycin) at 37°C for 3 days. Thereafter, OHC were transferred to serum-free medium (50% MEM-Hanks, 25% HBSS, 17 mM HEPES, 5 mM glucose, 1 mM L-glutamin, 25% Neurobasal-A, 0.5% B27, 0.5% gentamycin) for 14 days at 37°C in 5% CO_2_. For pre-selection, propidium iodide (PI; 2 μM) was added to the OHC 12 hours prior to the experiments to exclude damaged OHC. For OGD, hippocampal slices were transferred to six-well-plates with 1 mL of 10 mM mannitol-containing glucose-free Ringer solution. Cultures were placed in a gas-tight chamber at 37°C. Oxygen was replaced by 95% N_2_ and 5% CO_2_ for 10 minutes and slices were incubated for another 40 minutes. Untreated controls were maintained for the same time under a normoxic atmosphere in glucose-containing Ringer solution. After OGD, OHC were returned to normoxic culture conditions. Cell death was quantified as described previously [11].

### Co-cultivation of OHC with BM MNC and analysis of cellular damage

After OGD, slices were returned to normoxic standard culture conditions. For indirect co-cultivation 2.5x10E4 or 25x10E4 BM MNC were seeded under the interface cultures. After one and two days cell damage was determined by incubating OHC with PI (1 mg/mL) for two hours. Damage was analyzed in the cornu ammonis (CA1-CA2-CA3). To ensure comparability of data, fluorometric mean values obtained on day 1 were defined as 100%. All other data obtained are given as relative values.

### Experimental stroke and cell transplantation

The investigation conforms to the Guide for the Care and Use of Laboratory Animals published by the US National Institutes of Health (NIH Publication No. 85-23, revised 1996) and was approved by the appropriate regional authorities (reference number TVV18/07). In total, 33 male spontaneously hypertensive rats (SHR) at the age of 18 month (weighing 392 ± 32 g) were subjected to permanent middle cerebral artery occlusion as described previously [12]. The perioperative mortality amounted 36%. The remaining 21 SHR were randomly assigned to one of the following experimental groups: (1) transplantation of young BM MNCs, n = 7; (2) transplantation of old BM MNCs (n = 7); (3) injection of phosphate buffered solution (n = 7). Balanced randomization was performed by drawing lots. Exactly 24 h after stroke onset, 8x10E6 BM MNCs per kilogram bodyweight were transplanted intravenously via the tail vein. Accordingly, the control group received the same amount of vehicle solution at the same time. The application of cells or vehicle solution was performed by an investigator blinded to the group allocation.

### Behavioral tests

Neurofunctional deficits were quantified by a blinded investigator using two independent functional tests. At first, the modified Neurological Severity Score (mNSS) [13] was ascertained on the day before stroke as well as on days 2, 4 and 7 and weekly thereafter until day 63. The ladder rung test [14] was carried out the day before stroke and on days 2, 14, 28, 42 and 56. Briefly, the animals crossed a horizontal ladder with randomly mounted rungs spacing between 1 and 5 cm. Each run was videorecorded using three technical replicates and subsequently analyzed for each step of each limb separately. In doing so, the total number of steps and the total number of paw placing errors was ascertained and averaged for an animal at one time point.

### Magnetic resonance imaging

Lesion development was examined in vivo using magnetic resonance imaging (MRI; 1.5 T Gyroscan Intera human whole-body spectrometer equipped with a small loop RF-Coil (47 mm), Philips) on days 1, 8, 29 and 60. T2- weighted sequences (T2-TSE) were performed at each MRI session consisting of 20 transverse slices (matrix: 224 × 224; field of view: 50 mm; slice thickness: 1 mm). Corrected lesion volume was calculated by a blinded investigator as described previously [15] and expressed as the percentage of the day 1 infarct volume.

### Statistical analysis

Data obtained from in vitro experiments, slices cultures and MR investigations was first analyzed for normal distribution using the Shapiro-Wilk test. According to the distribution of data, unpaired two-tailed t-test or Mann-Whitney U test was used to analyze statistical differences between two groups. More than two groups were analyzed by one-way ANOVA or one-way ANOVA on ranks followed by Holm-Sidak or Turkey´s post hoc test. Time series data obtained from behavioral tests were summarized as area under the curve (AUC) [16] integrating all times points later than day 2. The AUCs were analyzed by multivariate regression models treating the usage of young and old bone marrow and the performance on day 2 as independent variables. The latter variable is included in view of the large impact of the initial lesion with respect to the outcome. A p-value of 0.05 or less was considered statistically significant. All data are shown as mean ± SD.

## Results

### Age-related changes in cell functionality

**Figure 1:**
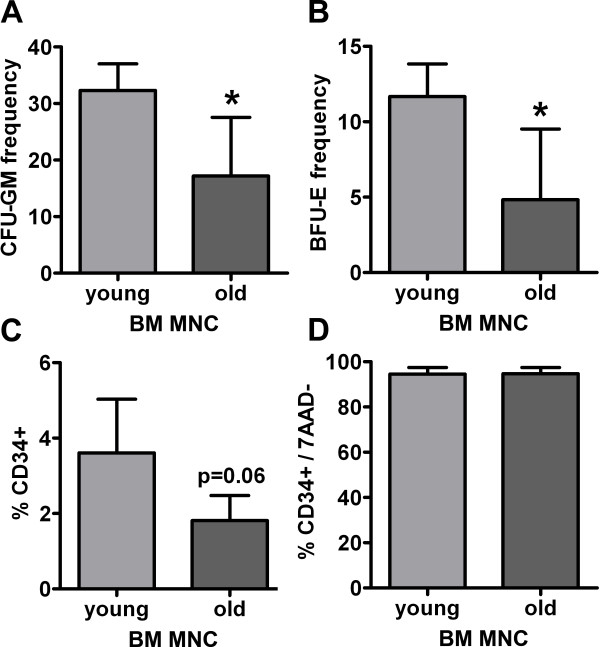
**A-C. Hematopoietic potential of human BM MNCs derived from young and old donors.** We found a statistically significant higher hematopoietic potential of young BM MNCs both in the investigation of colony forming units for granulocytes and macrophages (**A**; CFU-GM) and of erythroid burst forming units (**B**; BFU-E). This observation was supported by a 2-fold decrease of CD34+ hematopoietic stem cells in old BM MNCs (**C**). The vitality of CD34+ cells in both groups was above 90% and did not differ age-dependently (**D**). Values are means ± SD for 4 samples, each comprising three technical replicates, per group. *p < 0.05, ^§^p = 0.063 by t-test.

In the first set of experiments we examined whether age has a significant impact on the functional characteristics of BM MNCs. To answer this question the capacity of BM MNCs to generate hematopoietic colonies was investigated. The ability of BM MNCs derived from young donors with an average age of 24 years to form erythroid burst forming units (BFU-E) and granulocyte/monocyte forming units (CFU-GM) was significantly higher than that from donors with an average age of 68 years (Figure 1A-B). The flow cytometric analysis evidenced a 2-fold lower (albeit not statistically significant, p = 0.063) amount of CD34+ hematopoietic stem cells in BM MNCs of old donors (Figure 1). Vitality of CD34+ cells was above 90% and did not differ between both age groups (Figure 1D).

### Neuroprotective effects of BM MNC in vitro are age-dependent

**Figure 2:**
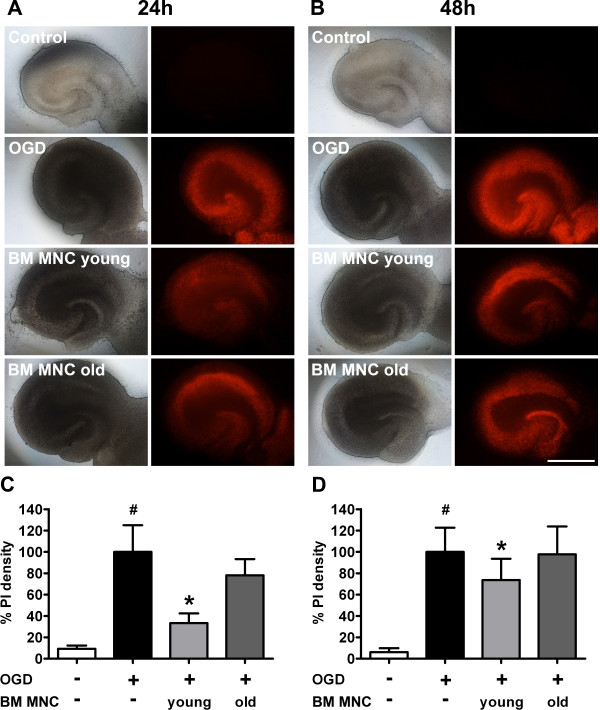
**A-D. Effects of BM MNC co-cultivation on hippocampal cell death after oxygen glucose deprivation (OGD). A** and **B** show representative illustrations of rat hippocampal slices (left column: light microscopic micrographs; right column: red Propidium iodide (PI) fluorescence). Compared to untreated control slices, the reduction of oxygen and glucose supply caused a significant increase of cell death (indicated by increased PI incorporation) at 24 h and 48 h (**C** and **D**: ^#^p < 0.001 by ANOVA). Thereby, PI positive cells occurred primarily within the cornu ammonis of the hippocampus (A and B). The co-cultivation with BM MNCs derived from young donors effected a significant reduction of PI-positive death cells at both investigation time points (*p < 0.001 by ANOVA). The addition of old BM MNCs did not significantly affect the number of PI-positive cells. Values are means ± SD for 10 samples per group. Scale bar: 2 mm.

To determine and compare the neuroprotective characteristics of old and young BM MNCs, we co-cultivated BM MNCs with rat hippocampal slices subjected to oxygen-glucose deprivation (OGD). PI staining was performed to monitor ischemia induced neural cell damage. Control slices showed a low incidence of PI incorporating cells, indicating a low rate of cell death. However, slices subjected to OGD showed a significant increase of cell death both after 24 h and 48 h (Figure 2). The co-cultivation of injured hippocampal slices with young BM MNCs resulted in a significant decrease of cell death at both investigation time points. This effect was, however, not detectable for aged BM MNCs (Figure 2).

### Aged hypertensive rats do not benefit from BM MNC transplantation

**Figure 3:**
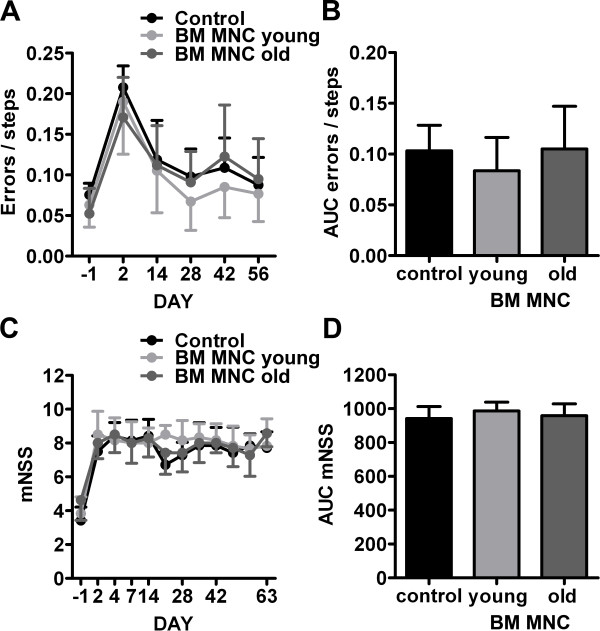
**A-D. Development of functional deficits measured by ladder rung (A-B) and neurological severity score (mNSS; C-D).** Experimental stroke caused a significant increase of ladder rung gait errors (**A**) and mNSS scores (**C**) at day 2. Gait errors decreased to a plateau during the remaining experiment, while the animals did not show any recovery of mNSS scores. The area under the curve (AUC) analyses (B and D) did not show any statistically significant differences between the experimental groups. Values are means ± SD for 7 samples per group.

The influence of recipient age and comorbidities on the efficacy of BM MNC transplantation after ischemic stroke can only be examined in vivo. Consequently, we performed a long-term experiment with aged spontaneously hypertensive rats. The efficacy of BM MNC transplantation was continuously monitored by two functional tests and by repeated measurement of the infarct volume using MRI. The ladder rung test showed a 4-fold increase of overall gait errors directly after the induction of experimental brain ischemia (Figure 3A). The frequency of gait errors decreased over two weeks following stroke, and remained at a stable plateau until the end of the experiment. The area under the curve (AUC) analysis exhibited a cumulated gait error rate of 8-10%. The AUC was clearly influenced by the performance on day 2 (i.e. the severity of infarct, beta = 0.5, p < 0.001), but was not affected by BM MNC transplantation (young BM MNC: beta = -0.01, p = 0.45, old BM MNC: beta = 0.02, p = 0.16, see Figure 3B). As for the ladder rung test, we found a clear increase of mNSS scores (i.e. an increase of neurological deficits) on day 2 after onset of brain ischemia. However, contrary to the ladder rung test, we found no signs for spontaneous recovery of mNSS scores during the experimental period (Figure 3C). Again, the AUC analysis showed no significant impact of BM MNC transplantation on post-stroke sensorimotor recovery (young BM MNC: beta = 40, p = 0.33, old BM MNC: beta = 16, p = 0.66, see Figure 3D).

**Figure 4:**
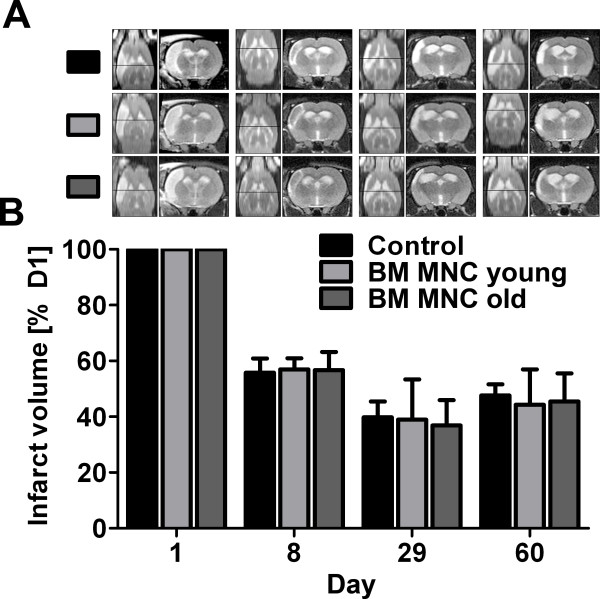
**A-B Determination of the infarct volume by magnetic resonance imaging (MRI).** Representative illustrations of T2 weighted MR sequences on days 1, 8, 29 and 60 after stroke induction (left: transversal view; right: coronal view). On the first day after stroke, all animals exhibited a circumscribed T2 hyperintensity within the supply territory of the middle cerebral artery. The further development of the ischemic lesion was characterized by a decrease of the brain edema within the first week, and an increasing organization with scar formation over time (**A**). The development of lesion volume did not differ significantly between any of the experimental groups (**B**). Values are means ± SD for 7 samples per group.

In the MR investigations, all animals showed cortical T2-hyperintensities that were conformable with an ischemic lesion in the supply territory of the middle cerebral artery (Figure 4A). Before cell transplantation or application of vehicle solution, animals featured comparable lesion volumes (control: 249 ± 30 mm^3^; young BM MNC: 207 ± 30 mm^3^; old BM MNC: 229 ± 36 mm^3^). To compensate inter-individual differences in stroke occurrence, we calculated the infarct volumes on days 8, 29 and 60 as percentage of day 1 infarct volume. The lesion volume decreased significantly within the first week after stroke and remained stable until the end of the experiment (Figure 4B). Transplantation of both young and old BM MNCs did not affect the lesion development compared to the control group (Figure 4B).

## Discussion

The question addressed by the present study was whether age influences the therapeutic concept of autologous BM MNC transplantation after acute stroke. The Framingham Study clearly demonstrated the relevance of age and high blood pressure for the lifetime risk of stroke [9] indicating the need to mimic these risk factors in preclinical stroke studies. Since BM MNCs offer the particular advantage of acute and autologous transplantability, age might influence both the patients’ susceptibility to and the functionality of the BM MNC graft.

By comparing the neuroprotective properties of human BM MNCs from young (24 ± 4 years) with aged (68 ± 1 years) donors in a cell culture model of cerebral hypoxia we found that young BM MNCs significantly suppress hippocampal cell death after oxygen glucose deprivation (OGD). This finding is in accordance with another recent in vitro study that revealed an attenuated amount of apoptotic neurons after hypoxic injury and cotreatment with BM MNC-derived supernatants [17]. The authors discussed the release of trophic factors and the modulation of microglia as contributing factors to the observed neuroprotective effects.

In our hands, aged BM MNCs did not show neuroprotective capabilities in the hippocampal OGD model. A BM MNC subpopulation, the bone marrow derived mesenchymal stromal cells (BMSC) are supposed to account for antiapoptotic effects after ischemic damage [18,19]. However, several studies revealed a decline in BMSC numbers and fitness with age. Thus it is tempting to speculate that senescent BM MNCs lack neuroprotective effects, as shown in the present study, due to an age-related drop in BMSC frequency and functionality [20].

In a second step, we evaluated the therapeutic efficacy of intravenously transplanted young and aged BM MNCs in aged hypertensive rats (SHR). Both BM MNCs from juvenile and elderly donors failed to decrease the lesion volume after experimental brain ischemia. Beyond that, functional recovery was not improved over a period of 56 days. These findings are, at least partly, in clear contrast to recent stroke studies demonstrating significant benefits of BM MNC transplantation in young and middle-aged (12 months old) healthy animals [5,6,8]. The differing results might be explainable by age and comorbid status of the laboratory animals used in this study. Aged individuals show a dysregulated cellular and genetic response to cerebral ischemia that finally determines a poor neurofunctional recovery. More precisely, augmented apoptosis and massive microglia activation that induces an enhanced inflammatory response account for accelerated infarct development in the aged brain [21,22]. Likewise, stroke in senescent rats is associated with a greater degree of oxidative cellular injury [23]. Another contributing factor might be the exacerbated astrocytic reaction in geriatric stroke rats that, in turn, impairs neurite outgrowth [24]. One of the cellular mechanisms of neuronal plasticity after stroke is axonal sprouting. Interestingly, aging delays the expression of growth-promoting genes during the sprouting response while growth-inhibitory genes are induced at earlier stages than in its younger counterparts [25]. Of note, enhancement of endogenous plasticity mechanisms such as axonal sprouting and synaptogenesis, amongst others, is considered to be a critical mode of action for cell-based therapies [26]. Thus, the temporal mismatch of growth-associated gene expression in the aging brain may interfere with the plasticity-related effects of cell therapy. Most importantly, the age-related shift of pathophysiological sequences in stroke might require adjustments of the therapeutic setting such as a prolonged treatment period or higher dosages of BM MNCs.

Moreover, previous studies revealed that the final infarct volume in SH rats develops rapidly within 1 h after the onset of permanent ischemia [27]. Thus, the lack of secondary infarct expansion in SH rats might additionally account for the absence of neuroprotective effects in this study. Unfortunately, our study is limited by the exclusive use of aged spontaneously hypertensive rats without controlling the age factor. Thus, the discrimination between the influence of hypertension, including its substantial impact on the cerebral microvessel system [28], and age is not possible. On the other hand, the combination of age, significant cardiovascular comorbidities, and a relatively advanced ischemic lesion development (sub-acute stage) describes the population of stroke patients which would primarily be eligible for BM MNC treatment in upcoming clinical trials.

Cell storage and processing may further influence the therapeutic potential of BM MNC. The cells used in our study had been cryopreserved prior to use, while the majority of positive therapeutic results were obtained in studies using freshly prepared BM MNC. Cryopreservation does not seem to affect the hematopoietic potential [29]. However, it is unclear whether freezing and/or thawing alters potential neuroprotective capabilities, which probably depend on completely different physiological processes. In fact, it has been shown that cryopreservation affects BM MNC physiology. Even single freezing-thawing cycles can enhance intrinsic proteolytic activity leading to the cleavage of apoptosis-related proteins [30], even though the impact of this alteration (and potentially others) on neuroprotection is unknown. Thus, thorough research is needed to elucidate this impact.

Another limitation of our study is the transplantation of human BM MNCs to rodents without an immunosuppressive treatment. The absence of beneficial effects in aged SHRs might be simply a consequence of xenograft rejection. However, recent animal stroke studies described significantly improved recovery by BM MNCs, albeit the transplanted cells died immediately after injection [5,31]. It is not conclusively elucidated so far, if and how long transplanted cells must survive to facilitate beneficial effects. In line with this, Thum and colleagues introduced the “dying stem cell hypothesis”. They suppose that apoptotic, rather than viable cells are responsible for the functional restoration after stem cell transplantation in ischemic injury via modulating the local immune response [32]. A further decisive reason why we desisted from immunosuppression is that the commonly applied agents may distort the value of this study by their neuroprotective properties [33] and by impacting the immunological processes that ultimately determine progress and outcome after stroke [34].

Although not investigated in the present study, several further mechanisms by which BM MNCs might improve recovery after ischemic stroke are age-dependent. It has been shown that young BM MNCs promote neovascularization in models of limb ischemia [35] myocardial infarction [36] and ischemic white matter damage [37]. However, aging impairs the angiogenic capacity of BM MNCs [38], a finding that is in agreement with an impaired VEGF production and migratory response to VEGF in aged BM MNCs [39]. Furthermore, it has been shown that vasculogenesis after BM MNC transplantation is dependent on the CD34+ cell fraction [40] and that CD34+ cells enhance post-ischemic neurogenesis [41]. Our finding that the frequency of hematopoietic progenitor cells is significantly reduced in senescent BM MNCs might therefore compromise the therapeutic efficacy of self-donated BM MNCs in aged patients.

## Conclusions

In summary, a neuroprotective effect of young, but not of senescent BM MNCs in an in vitro model of cerebral ischemia was shown. Aged hypertensive rats, however, did not benefit from acute BM MNC treatment, regardless of the donor’s age. Age and comorbidity should thus be taken into consideration when studying the efficacy of autologous cell transplantation for stroke.

## Competing interests

The authors declare that they have no competing interests.

## Authors’ contributions

The manuscript was written by DCW, GW and MP. DCW, JB, MP, AKa, DH, MS, AKr and GW designed the study and supervised the experiments. In vitro and ex vivo investigations were performed by MP, animal experiments were conducted by MB and ML. CV analyzed the MR sequences. DH and MS analyzed data obtained from behavioral tests. All authors have read and approved the final version of the manuscript.

## References

[B1] Barber PA Zhang J Demchuk AM Hill MD Buchan AM Why are stroke patients excluded from TPA therapy? An analysis of patient eligibility Neurology 2001 56 1015 1020 10.1212/WNL.56.8.1015 11320171

[B2] Chopp M Li Y Zhang ZG Mechanisms underlying improved recovery of neurological function after stroke in the rodent after treatment with neurorestorative cell-based therapies Stroke 2009 40 S143 S145 10.1161/STROKEAHA.108.533141 19064763 PMC2854491

[B3] Savitz SI Misra V Launching intravenous bone marrow cell trials for acute stroke Regen Med 2009 4 639 641 10.2217/rme.09.41 19761387

[B4] Savitz SI Misra V Kasam M Juneja H Cox CS Jr Alderman S Aisiku I Kar S Gee A Grotta JC Intravenous autologous bone marrow mononuclear cells for ischemic stroke Ann Neurol 2011 70 59 69 10.1002/ana.22458 21786299

[B5] Brenneman M Sharma S Harting M Strong R Cox CS Jr Aronowski J Grotta JC Savitz SI Autologous bone marrow mononuclear cells enhance recovery after acute ischemic stroke in young and middle-aged rats J Cereb Blood Flow Metab 2010 30 140 149 10.1038/jcbfm.2009.198 19773802 PMC2893568

[B6] Giraldi-Guimaraes A Rezende-Lima M Bruno FP Mendez-Otero R Treatment with bone marrow mononuclear cells induces functional recovery and decreases neurodegeneration after sensorimotor cortical ischemia in rats Brain Res 2009 1266 108 120 10.1016/j.brainres.2009.01.062 19368806

[B7] Yoshihara T Ohta M Itokazu Y Matsumoto N Dezawa M Suzuki Y Taguchi A Watanabe Y Adachi Y Ikehara S Sugimoto H Ide C Neuroprotective effect of bone marrow-derived mononuclear cells promoting functional recovery from spinal cord injury J Neurotrauma 2007 24 1026 1036 10.1089/neu.2007.132R 17600518

[B8] Iihoshi S Honmou O Houkin K Hashi K Kocsis JD A therapeutic window for intravenous administration of autologous bone marrow after cerebral ischemia in adult rats Brain Res 2004 1007 1 9 10.1016/j.brainres.2003.09.084 15064130

[B9] Seshadri S Beiser A Kelly-Hayes M Kase CS Au R Kannel WB Wolf PA The lifetime risk of stroke: estimates from the Framingham Study Stroke 2006 37 345 350 10.1161/01.STR.0000199613.38911.b2 16397184

[B10] Stoppini L Buchs PA Muller D A simple method for organotypic cultures of nervous tissue J Neurosci Methods 1991 37 173 182 10.1016/0165-0270(91)90128-M 1715499

[B11] Sarnowska A Braun H Sauerzweig S Reymann KG The neuroprotective effect of bone marrow stem cells is not dependent on direct cell contact with hypoxic injured tissue Exp Neurol 2009 215 317 327 10.1016/j.expneurol.2008.10.023 19063882

[B12] Riegelsberger UM Deten A Posel C Zille M Kranz A Boltze J Wagner DC Intravenous human umbilical cord blood transplantation for stroke: Impact on infarct volume and caspase-3-dependent cell death in spontaneously hypertensive rats Exp Neurol 2011 227 218 223 10.1016/j.expneurol.2010.11.008 21087606

[B13] Chen J Sanberg PR Li Y Wang L Lu M Willing AE Sanchez-Ramos J Chopp M Intravenous administration of human umbilical cord blood reduces behavioral deficits after stroke in rats Stroke 2001 32 2682 2688 10.1161/hs1101.098367 11692034

[B14] Metz GA Whishaw IQ Cortical and subcortical lesions impair skilled walking in the ladder rung walking test: a new task to evaluate fore- and hindlimb stepping, placing, and co-ordination J Neurosci Methods 2002 115 169 179 10.1016/S0165-0270(02)00012-2 11992668

[B15] Kranz A Wagner DC Kamprad M Scholz M Schmidt UR Nitzsche F Aberman Z Emmrich F Riegelsberger UM Boltze J Transplantation of placenta-derived mesenchymal stromal cells upon experimental stroke in rats Brain Res 2010 1315 128 136 20004649 10.1016/j.brainres.2009.12.001

[B16] Matthews JN Altman DG Campbell MJ Royston P Analysis of serial measurements in medical research BMJ 1990 300 230 235 10.1136/bmj.300.6719.230 2106931 PMC1662068

[B17] Sharma S Yang B Strong R Xi X Brenneman M Grotta JC Aronowski J Savitz SI Bone marrow mononuclear cells protect neurons and modulate microglia in cell culture models of ischemic stroke J Neurosci Res 2010 88 2869 2876 20629187 10.1002/jnr.22452 PMC3401573

[B18] Chopp M Li Y Treatment of neural injury with marrow stromal cells Lancet Neurol 2002 1 92 100 10.1016/S1474-4422(02)00040-6 12849513

[B19] Tate CC Fonck C McGrogan M Case CC Human mesenchymal stromal cells and their derivative, SB623 cells, rescue neural cells via trophic support following in vitro ischemia Cell Transplant 2010 19 973 984 10.3727/096368910X494885 20350349

[B20] Stolzing A Jones E McGonagle D Scutt A Age-related changes in human bone marrow-derived mesenchymal stem cells: consequences for cell therapies Mech Ageing Dev 2008 129 163 173 10.1016/j.mad.2007.12.002 18241911

[B21] Popa-Wagner A Badan I Walker L Groppa S Patrana N Kessler C Accelerated infarct development, cytogenesis and apoptosis following transient cerebral ischemia in aged rats Acta Neuropathol 2007 113 277 293 10.1007/s00401-006-0164-7 17131130

[B22] Badan I Buchhold B Hamm A Gratz M Walker LC Platt D Kessler C Popa-Wagner A Accelerated glial reactivity to stroke in aged rats correlates with reduced functional recovery J Cereb Blood Flow Metab 2003 23 845 854 12843788 10.1097/01.WCB.0000071883.63724.A7

[B23] Li S Zheng J Carmichael ST Increased oxidative protein and DNA damage but decreased stress response in the aged brain following experimental stroke Neurobiol Dis 2005 18 432 440 10.1016/j.nbd.2004.12.014 15755669

[B24] Popa-Wagner A Dinca I Yalikun S Walker L Kroemer H Kessler C Accelerated delimitation of the infarct zone by capillary-derived nestin-positive cells in aged rats Curr Neurovasc Res 2006 3 3 13 10.2174/156720206775541732 16472121

[B25] Li S Carmichael ST Growth-associated gene and protein expression in the region of axonal sprouting in the aged brain after stroke Neurobiol Dis 2006 23 362 373 10.1016/j.nbd.2006.03.011 16782355

[B26] Bliss TM Andres RH Steinberg GK Optimizing the success of cell transplantation therapy for stroke Neurobiol Dis 2010 37 275 283 10.1016/j.nbd.2009.10.003 19822211 PMC2818270

[B27] Legos JJ Lenhard SC Haimbach RE Schaeffer TR Bentley RG McVey MJ Chandra S Irving EA Andrew AP Barone FC SB 234551 selective ET(A) receptor antagonism: perfusion/diffusion MRI used to define treatable stroke model, time to treatment and mechanism of protection Exp Neurol 2008 212 53 62 10.1016/j.expneurol.2008.03.011 18462720

[B28] Pantoni L Cerebral small vessel disease: from pathogenesis and clinical characteristics to therapeutic challenges Lancet Neurol 2010 9 689 701 10.1016/S1474-4422(10)70104-6 20610345

[B29] Nicol A Nieda M Donaldson C Denning-Kendall P Truman C Bradley B Hows J Cryopreserved human bone marrow stroma is fully functional in vitro Br J Haematol 1996 94 258 265 10.1046/j.1365-2141.1996.d01-1812.x 8759884

[B30] Schmidt-Mende J Hellström-Lindberg E Joseph B Zhivotovsky B Freezing induces artificial cleavage of apoptosis-related proteins in human bone marrow cells J Immunol Methods 2000 245 91 94 10.1016/S0022-1759(00)00285-4 11042286

[B31] Yang B Strong R Sharma S Brenneman M Mallikarjunarao K Xi X Grotta JC Aronowski J Savitz SI Therapeutic time window and dose response of autologous bone marrow mononuclear cells for ischemic stroke J Neurosci Res 2011 89 833 839 10.1002/jnr.22614 21412816 PMC3412881

[B32] Thum T Bauersachs J Poole-Wilson PA Volk HD Anker SD The dying stem cell hypothesis: immune modulation as a novel mechanism for progenitor cell therapy in cardiac muscle J Am Coll Cardiol 2005 46 1799 1802 10.1016/j.jacc.2005.07.053 16286162

[B33] Sullivan PG Sebastian AH Hall ED Therapeutic window analysis of the neuroprotective effects of cyclosporine A after traumatic brain injury J Neurotrauma 2011 28 311 318 10.1089/neu.2010.1646 21142667 PMC3037811

[B34] Chamorro A Meisel A Planas AM Urra X van de Beek D Veltkamp R The immunology of acute stroke Nat Rev Neurol 2012 8 401 410 10.1038/nrneurol.2012.98 22664787

[B35] Shintani S Murohara T Ikeda H Ueno T Sasaki K Duan J Imaizumi T Augmentation of postnatal neovascularization with autologous bone marrow transplantation Circulation 2001 103 897 903 10.1161/01.CIR.103.6.897 11171801

[B36] Zhang S Guo J Zhang P Liu Y Jia Z Ma K Li W Li L Zhou C Long-term effects of bone marrow mononuclear cell transplantation on left ventricular function and remodeling in rats Life Sci 2004 74 2853 2864 10.1016/j.lfs.2003.10.035 15050423

[B37] Fujita Y Ihara M Ushiki T Hirai H Kizaka-Kondoh S Hiraoka M Ito H Takahashi R Early Protective Effect of Bone Marrow Mononuclear Cells Against Ischemic White Matter Damage Through Augmentation of Cerebral Blood Flow Stroke 2010 41 2938 2943 10.1161/STROKEAHA.110.596379 20947840

[B38] Zhuo Y Li SH Chen MS Wu J Kinkaid HY Fazel S Weisel RD Li RK Aging impairs the angiogenic response to ischemic injury and the activity of implanted cells: combined consequences for cell therapy in older recipients J Thorac Cardiovasc Surg 2010 139 1286 1294 10.1016/j.jtcvs.2009.08.052 19931095

[B39] Sugihara S Yamamoto Y Matsuura T Narazaki G Yamasaki A Igawa G Matsubara K Miake J Igawa O Shigemasa C Hisatome I Age-related BM-MNC dysfunction hampers neovascularization Mech Ageing Dev 2007 128 511 516 10.1016/j.mad.2007.06.009 17688912

[B40] Iwasaki H Kawamoto A Ishikawa M Oyamada A Nakamori S Nishimura H Sadamoto K Horii M Matsumoto T Murasawa S Shibata T Suehiro S Asahara T Dose-dependent contribution of CD34-positive cell transplantation to concurrent vasculogenesis and cardiomyogenesis for functional regenerative recovery after myocardial infarction Circulation 2006 113 1311 1325 10.1161/CIRCULATIONAHA.105.541268 16534028

[B41] Taguchi A Soma T Tanaka H Kanda T Nishimura H Yoshikawa H Tsukamoto Y Iso H Fujimori Y Stern DM Naritomi H Matsuyama T Administration of CD34+ cells after stroke enhances neurogenesis via angiogenesis in a mouse model J Clin Invest 2004 114 330 338 15286799 10.1172/JCI20622 PMC484977

